# Determination of the Awareness about and Need for Health Support Pharmacies as the Provider of Consultation Service about Nutrition Education and Diet-Related Health Promotion by Health Professionals in Japan

**DOI:** 10.3390/nu14010165

**Published:** 2021-12-30

**Authors:** Tsuyoshi Chiba, Nanae Tanemura, Chiharu Nishijima

**Affiliations:** Department of Food Function and Labeling, National Institute of Health and Nutrition, National Institutes of Biomedical Innovation, Health and Nutrition, Tokyo 162-8363, Japan; n-tanemura@nibiohn.go.jp (N.T.); c-nishijima@nibiohn.go.jp (C.N.)

**Keywords:** health support pharmacy (HSP), health food, pharmacist, dietician

## Abstract

Health support pharmacies (HSPs) have been established as a new category of pharmacies in Japan. In addition to prescriptions, HSPs provide several health services, including consultations on diet/nutrition, health foods, and nursing care. Therefore, not only individuals receiving medications but also community residents should have access to HSPs. However, it is unclear whether people are aware of HSPs. Thus, the purpose of this study was to assess the awareness about HSPs and determine the need for their services. To this end, we conducted an online cross-sectional questionnaire survey in 10,000 Japanese adults. Approximately 60.2% of the participants were aware of family pharmacies/pharmacists, and 21.8% of these participants had a family pharmacy/pharmacist. Meanwhile, 2.6% of the participants were aware of HSPs, while 9.2% of the participants had only heard of HSPs. Awareness of HSPs was higher among men and younger individuals than among women and older generations. In addition, only 7.2% of the participants were aware of the location of the HSP in their area of residence. At the time at which this survey was conducted, only 3.5% of the participants were using HSP services, and half of them did not perceive the merits of using these services. However, 44.4% of the participants wished to avail themselves of HSP services in the future, and this desire increased with age. Half of the participants wished to use services that were associated with drugs, and the need for other services, such as consultations on diet/nutrition or health foods, was low. In conclusion, there was low awareness about HSPs among the survey participants. However, from our findings, we gathered that if individuals are aware of HSPs, they will wish to use HSP services. To improve healthy life expectancy, it is important to increase awareness about HSPs and their number.

## 1. Introduction

Health foods (also referred to “dietary supplements”) have been touted as a means of self-care for managing health. Especially vitamins and minerals are helpful to keep our health. However, various health foods, especially herbal products, are in the markets, and some of them claim health benefits with scientific evidence, but others claim health benefits without scientific evidence [1]. In this situation, inappropriate health food use has been reported among consumers, such as simultaneous intake of multiple products and use for therapeutic purposes. In addition, a portion of consumers have developed health problems that are thought to be caused by the use of health foods, and currently, health foods are not being used appropriately for self-care [2,3,4]. Further, even though concomitant use of medicines and health foods not only adversely affects the therapeutic effect due to interactions between medicine and health foods but also may cause health issues [5,6], some patients use them concomitantly [7,8]. Therefore, medical professionals need to pay attention to the use of health foods by patients. However, if the attitude toward health food use is negative among healthcare professionals, patients may not inform their physicians and pharmacists about such use. In a previous survey, only 30% of the participants who used health foods consulted their doctors or pharmacists about the issue [7,8]. One of the reasons given by the participants for this lack of consultation was that they believed their doctors and pharmacists would reject such use without any explanations. Nevertheless, the use of health foods has been found to motivate patients, especially cancer patients, to recover from their illnesses quickly [9]. Almost half of cancer patients used health foods as alternative medicines that were not authorized. Moreover, health foods such as vitamins and minerals are considered beneficial for patients, especially in cancer patients, when they lack appetite due to the illness [10]. In addition, malnutrition or deficiency of some vitamin or mineral is one of the risks for infection and severity of COVID-19 [11,12], and a lot of clinical trials are ongoing. Therefore, it is important to establish an environment in which health foods can be used appropriately.

Japan is about to become a super-aged society. In October 2020, 28.8% of Japan’s population was aged ≥65 years. Furthermore, by 2065, one in every 2.6 people will be aged ≥65 years, and one in every 3.9 people will be aged ≥75 years [13]. The Strategy for the Revitalization of Japan includes the following: “Pharmacies should be community-based health information centers that provide advice on the proper use of over-the-counter (OTC) drugs and other products, as well as health-related consultation and information, and use of pharmacies and pharmacists should be encouraged for the promotion of self-medication.” In this regard, Health support pharmacies (HSPs) were established based on that statement of the Japan Revitalization Strategy [14].

HSPs were established as part of the efforts to create an environment for total care of not only patients but also community residents, including appropriate health food use in Japan. The definition of HSPs includes two functions: first, a primary-care function, such as the centralized management of prescriptions, 24-h availability, home services, and coordination with medical and long-term care facilities; second, a health-support function, such as nutrition education, diet-related health promotion, and health food usage for disease prevention, targeting the general public. HSPs were institutionalized on 1 April 2016 [15]. In addition to offering the basic services of a conventional family pharmacy (same as community pharmacy in other countries), such as providing an integrated understanding of medication information, pharmacological management, and guidance based on that information, HSPs are meant to serve as pharmacies where patients can feel free to seek consultation about not only medication, which include OTC medications, but also diet, nutrition, health food, and nursing care. These pharmacies are expected to actively support the health of not only patients but also local community residents. This study was aimed at evaluating the level of awareness about HSPs and determining the services expected from these institutions at a nationwide level in Japan.

## 2. Methods

### 2.1. Study Participants

An online questionnaire survey was administered to men and women between the ages of 20 and 89 years who were registered as monitors with Cross Marketing Inc. (Tokyo, Japan), which is one of companies that conducts various surveys, including an internet surveillance. These companies have their original respondent panels as monitors in each surveillance. Specifically, an email containing the purpose of the questionnaire and the URL of the questionnaire response site was sent to the email addresses registered by the members, and the survey was conducted until 10,000 responses were collected (13–14 July 2020), with equal distribution of sex and age. A request for cooperation in this study and a statement that a response to the questionnaire would be considered as consent for cooperation in this study were included on the website for the questionnaire response. This informed consent form was approved by the Research Ethics Committee of the National Institutes of Biomedical Innovation, Health and Nutrition. Personal information and privacy protection were contracted between the survey participants and Cross Marketing Inc. Cross Marketing Inc. has 2 million monitors who are publicly solicited and is taking measures to prevent duplicate and fraudulent registrations through trap surveys and irregular mandatory updates of registration information.

### 2.2. Survey Contents

Questions pertaining to the following items were asked: awareness and use of family pharmacies/pharmacists and HSPs, need for each service provided at HSPs, qualified personnel with whom participants wish to consult, medication status, use of health foods, and concerns about daily dietary habits. In addition, data on participants’ sex and age were obtained from the survey company’s registration data.

### 2.3. Statistical Analysis

Descriptive statistical analyses were performed for all data, and the results are presented as percentages (%). Differences in distribution among groups were compared using the chi-squared (*χ*^2^) test. All statistical analyses were performed using Cross Finder 2 ver.2.3.2.0 provided by the internet survey company (Cross Marketing Inc., Tokyo, Japan), and a *p*-value of <0.05 was considered statistically significant.

## 3. Results

### 3.1. Attributes of the Respondents

The total number of respondents was 10,000, of which 5000 were male and 5000 were female; 2000 were in each age group: 20s, 30s, 40s, 50s, and over 60s. The 2000 respondents aged 60 years or older were further divided into the following two age groups: 60–74 years (early-stage elderly participants: 1820) and ≥75 years (late-stage elderly participants: 180).

### 3.2. Awareness and Use of Family Pharmacy/Pharmacist Services and HSPs

Family pharmacy/pharmacist services were used by 21.8% of the respondents; 60.2% of the respondents had knowledge of family pharmacies/pharmacists, including those who knew about them but did not avail themselves of those services (Table 1). Although there was no sex difference in the number of users, more women (25.2%) than men (19.7%) had knowledge of the services among those who did not use them. In terms of age, the percentage of those who used the service increased with age, with 47.8% of the late-stage elderly (≥75 years) using the service.

On the other hand, only 2.6% of the respondents had knowledge of HSPs, and 9.2% had heard of them to some extent, indicating that approximately 90% of the respondents did not know about HSPs (Table 2). Male respondents (3.3%) showed a higher level of awareness than did the female respondents (2.0%), and the level of awareness tended to decrease as the age of the respondents increased. The respondents were asked whether there was an HSP in their area of residence; 2.4% of the respondents answered that there was one within walking distance, 2.4% of the respondents answered that there was one within cycling distance, 2.6% answered that there was one within driving distance, and 1.8% answered that there was one on the way to or nearby school/workplace. The majority (92.8%) answered that there was no HSP within the area of residence or that they did not know if there was any.

### 3.3. Status of Medication and Use of Health Foods

Regarding medication status, 35.9% of the total respondents answered that they were taking prescription medications, and 13.6% answered that they were taking OTC medications (Table 3). The number of those taking any drug increased with age, with the percentage being 33.9% among those in their 20s and 75.6% among those were aged ≥75 years. The percentage of those taking prescription drugs tended to increase with age, while the percentage of those taking OTC drugs tended to decrease with age. With respect to the use of health food products, 22.2% of the respondents used vitamin/mineral products, 20.9% used other products that included herbs and pre/probiotics, and 36.8% used one or both products (Table 4). The prevalence of vitamin and mineral product use did not differ by age. However, the prevalence of other product use tended to increase with age, with the percentages being 14.1%, 27.9%, and 27.8% among those aged 20–29, 60–74, and ≥75 years, respectively.

### 3.4. Awareness of Daily Dietary Habits

When asked about concerns pertaining to daily dietary habits, the respondents’ answers were as follows: undernourishment, 28.1%; overeating, 23.0%; unbalanced diet, 31.1%; and small appetite, 4.8% (Table 5). Some of them were receiving nutritional guidance at hospitals or health centers (3.0%). Overall, compared with the older respondents, the younger ones tended to be more anxious about their daily dietary habits.

### 3.5. Need for HSPs

HSPs not only provide medication guidance but also offer guidance on the use of health foods, daily eating habits, and nutrition. Respondents were asked to read a description of the services available at HSPs and then asked about their need for an HSP. Overall, 1.7%, 1.8%, and 44.4% of the respondents answered that they use HSPs, they use them but do not feel they are beneficial, and they have not used them but would like to use them in the future, respectively. However, half of the respondents (52.2%) answered that they did not currently use the service and would not like to use it in the future (Table 6). In terms of age, the number of respondents who already used the service was higher in the younger age group and decreased as the age group increased, while the percentage of respondents who wanted to use the service in the future increased as the age group increased.

### 3.6. Need for Individual Services Provided by HSPs

We asked the respondents about the needs for each service available at the HSPs; the prevalence of services that were being using was only 0.7% to 2.2% in each. The most frequent responses for future use were as follows: explanation and combination of prescription drugs (52.0%); prescription of drugs (51.0%); side effects (48.9%); and explanation and combination of OTC drugs (48.7%). Thus, there was high demand for services related to medication, and responses for consultations regarding dietary/nutritional guidance and health foods were limited to 30–40% (Table 7).

It is believed that pharmacists are, in principle, involved in handling services at each consultation in pharmacies. However, currently, some pharmacies have specialized staff such as dietitians and advisory staffs for health foods. After explaining the background of each qualification, we asked the respondents with whom they would like to consult for each service. The majority of respondents (61.9–64.6%) answered pharmacists for items related to medicines, and the majority of respondents answered dietitians (49.5%) for diet/nutrients (Table 8). However, more than 30% of the respondents answered that they were not concerned about the specialty of the professional.

When asked whether they knew of any pharmacies where they could consult about their daily diet and health foods, 13.6% of the respondents answered that they knew about one, and 16.6% answered that they knew of pharmacies with dietitians.

## 4. Discussion

In this study, we surveyed consumers’ awareness about HSPs and their service needs. The survey revealed that 11.8% of respondents were aware of HSPs. Although this result was slightly higher than the results of a survey of 1000 consumers conducted by the Japan Pharmaceutical Manufacturers Association (8.4%) in September 2018 and a survey of 1944 consumers conducted by the Cabinet Office (8.0%) in October 2020, about almost the same time as the present survey, all surveillances indicated that the awareness among consumers was still quite low. In addition, it has been reported that users of HSPs also avail themselves of these services without being aware that pharmacies that they used are HSPs [16]. The results of the present survey indicate that consumers have high expectations for the services provided by HSPs, although the awareness about them remains low. However, the expectations for each service, except for diet/nutrients, are biased toward pharmacists, and consumers’ understanding of other specialists’ services seems insufficient.

As in previous surveys, the results of the present survey indicate that the rate of prescription drug use increased with age and that the rate of using health food products other than vitamins and minerals increased. This suggests that as the respondents’ age increases, there is a possibility that many of them use both drugs and health foods concomitantly. In fact, in a previous survey, the combined use of medicines and health foods (concentrates used as supplements were surveyed) was approximately 20% among adults aged 20 years and higher [7]. Many respondents consumed multiple drugs and multiple health foods. In addition to the health hazards caused by health foods themselves, those caused by the concomitant use of health foods and drugs are paid attention. Numerous health foods, especially herbs such as St. John’s wort [17], ginkgo biloba extract [18], and aojiru (vegetable juice made from green leafy vegetables) [19], have been reported to have potential interactions with drugs [20,21,22]. On the other hand, warfarin is the drug that is most known to interact with food (including health foods), and many health problems have been reported due to its concomitant use [23]. Currently, various public organizations issue warnings about the concomitant use of medicines and health foods; however, unfortunately, these warnings do not seem to reach consumers (patients). Therefore, physicians and pharmacists need to pay attention to the use of health foods by patients. Basically, the best approach is to stop health food consumption if the patient is taking medicines; however, if the patient is flatly told to stop without their opinion being heard, the patient may keep using them without telling the doctor or pharmacist [2]. In such situations, there is a high possibility that the use of health foods will hinder appropriate treatment. Therefore, it is necessary to create an environment in which health foods can be used appropriately in consultation with medical professionals [24].

The number of HSPs has been steadily increasing since the system was established, with 2608 pharmacies registered as of June 2021. However, this number represents only roughly 4.3% of the approximately 60,000 pharmacies in Japan. In the present survey as well, only 7.2% of the respondents answered that there was an HSP in their area of residence. We collected both “I do not know if there is a health support pharmacy” and “There is none” as one option in this survey. Hence, it was not possible to distinguish whether there was no HSP nearby or whether the respondents did not know about it. However, the results still suggest that both the number of HSPs and awareness of HSPs are insufficient.

With the expansion of pharmacy’s services such as HSP, the knowledge required of pharmacists is also increasing. Consultations on diet, nutrition, and health foods are expected to increase. There are two possible ways to address this situation. The first is to improve the level of pharmacists’ knowledge by providing lectures on these topics at universities so that they can acquire basic knowledge for working in these settings in future. In this regard, it has been reported that even a single lecture can bring about a change in the knowledge of health foods [25]. Although a single lecture is not enough to enable the acquisition of sufficient knowledge, it is expected to change attitudes toward learning by changing awareness. The second is to staff each HSP with specialists, in addition to pharmacists, who can provide advice on various topics, such as dietitians for providing diet and nutrition advice and various advisory staff for providing advice on health foods. With the staffing of these qualified personnel, pharmacists can continue to hone their skills as drug experts and consult with each of the other respective specialists when necessary. However, even in the current survey, the needs for services other than dispensing of medications and drug consultations at HSPs remained low, and the future needs are unclear. Given this situation, it would be cost-prohibitive for each pharmacy to have these professionals on staff on a regular basis. However, as the needs of consumers increase, pharmacies are likely to respond to these needs. Hence, it will be necessary to resolve these issues to make HSPs hubs for community communication in the future.

The strength of this study is that this is the first report that clarifies the awareness of and need for health support pharmacies. In addition, this online survey was conducted with participants who live everywhere in Japan. On the other hand, a limitation is that this study was an online survey, so the participants were registrants of the survey company. In this regard, we have to carefully treat our data as general, even though internet and online questionnaires have become popular across all age groups. In addition, HSP is a unique system in Japan. However, health food use is increasing around the world, so the concept of HSP is also important in other countries.

## 5. Conclusions

There was low awareness about HSPs among the survey participants; almost 90% of them did not know about HSPs. However, from our findings, we gathered that if individuals were aware of HSPs, they would wish to use HSP services, as one third of them are interested in diet/nutrients and health foods. Furthermore, to improve healthy life expectancy, it is important to increase both awareness of HSPs and the number of them to help consumers easily find and access HSPs because more than 90% of participants answered that there was no HSP within their residence area or that they did not know if there was.

## Figures and Tables

**Table 1:** Recognition of family pharmacies/pharmacists.

	I Have a Family Pharmacy/Pharmacist.	I Know of Family Pharmacies/Pharmacists, but I Do Not Have One.	I Know of Family Pharmacies/Pharmacists, but I Do Not Have an Opportunity to Use One.	I Do Not Know Family Pharmacies/Pharmacists.	*p*-Value
Total	21.8	22.4	16.0	39.8	
Sex					<0.001
Male	21.1	19.7	16.1	43.2	
Female	22.5	25.2	15.9	36.4	
Age					<0.001
20s	15.6	22.5	19.9	42.1	
30s	15.8	22.0	18.5	43.7	
40s	18.4	23.7	16.2	41.9	
50s	25.3	22.7	14.7	37.4	
60–74 ^1^	32.5	21.7	10.9	34.9	
≥75 ^2^	47.8	17.2	10.0	25.0	

*n* = 10,000, ^1^ *n* = 1820 (Male: 906, Female: 914), ^2^ *n* = 180 (Male: 94, Female: 86). Differences between male and female or among each generation were examined using the chi-square (*χ*^2^) test.

**Table 2:** Recognition of health support pharmacy.

	I Know of Health Support Pharmacy.	I Have Merely Heard of Health Support Pharmacy, but I Do Not Know of It.	I Do Not Know of Health Support Pharmacy.	*p*-Value
Total	2.6	9.2	88.2	
Sex				<0.001
Male	3.3	10.3	86.4	
Female	2.0	8.0	90.0	
Age				<0.001
20s	4.7	11.5	83.9	
30s	2.9	9.1	88.1	
40s	2.1	8.8	89.2	
50s	1.9	7.8	90.4	
60–74 ^1^	1.5	8.4	90.2	
≥75 ^2^	2.8	12.2	85.0	

*n* = 10,000, ^1^ *n* = 1820 (Male: 906, Female: 914), ^2^ *n* = 180 (Male: 94, Female: 86). Differences between male and female or among each generation were examined using the chi-square (*χ*^2^) test.

**Table 3:** Statement of medication.

Age (Years)	I Take Prescription Drugs.	I Take OTC ^1^ Drugs.	I Do Not Take any Drugs.	*p*-Value
Total	35.9	13.6	55.4	
20s	23.4	15.1	66.1	<0.001
30s	24.3	16.2	64.3	
40s	30.7	15.4	59.3	
50s	41.5	12.4	50.6	
60–74	58.5	8.7	37.7	
≥75	73.9	10.0	24.4	

Differences among each generation were examined using the chi-square (*χ*^2^) test. ^1^ OTC: over-the-counter

**Table 4:** Prevalence of health food use.

Age (Years)	I Am Using Vitamin/Mineral Supplements.	I Am Using Non-Vitamin/Non-Mineral Supplements.	I Do Not Use Any Health Foods.	*p*-Value
Total	22.2	20.9	63.2	
20s	22.9	14.1	68.3	<0.001
30s	23.2	17.5	66.2	
40s	22.7	21.1	62.9	
50s	21.7	23.9	60.9	
60–74	20.8	27.9	58.0	
≥75	18.3	27.8	58.3	

Differences among each generation were examined using the chi-square (*χ*^2^) test.

**Table 5:** Concerns about dietary habits.

Age (Years)	Undernourishment	Overeating	Unbalanced Diet	Small Appetite	Consulting with Hospital/Health Center	Nothing	*p*-Value
Total	28.1	23.0	31.1	4.8	3.0	21.4	
20s	35.4	20.5	31.9	6.4	2.8	39.9	<0.001
30s	35.5	24.1	35.1	4.5	3.0	37.5	
40s	30.2	28.6	35.0	4.4	3.0	34.2	
50s	25.4	24.2	32.9	4.6	2.8	40.4	
60–74	14.7	18.0	21.4	3.9	3.4	54.0	
≥75	10.0	12.2	13.3	5.6	2.8	66.7	

Differences among each generation were examined using the chi-square (*χ*^2^) test.

**Table 6:** Needs for health support pharmacy.

Age (Years)	I Am Using Health Support Pharmacy, and I Want to Continue Using It.	I Am Using Health Support Pharmacy, but I Do Not Perceive the Merits.	I Do Not Use Health Support Pharmacy, but I Want to Use it.	I Do Not Use Health Support Pharmacy, and I Will Not Use It.	*p*-Value
Total	1.7	1.8	44.4	52.2	
20s	3.0	3.2	37.3	56.6	<0.001
30s	2.0	2.0	38.2	67.9	
40s	1.2	1.8	47.1	50.0	
50s	1.1	1.4	48.3	49.3	
60–74	1.0	0.4	51.0	47.6	
≥75	2.8	1.1	53.9	42.2	

Differences among each generation were examined using the chi-square (*χ*^2^) test.

**Table 7:** Needs for each service of health support pharmacy.

	I Am Using the Service.	I Do Not Use the Service, but I Want to Use it.	I Do Not Want to Use the Service.	I Do Not Know.
Prescription	2.2	51.0	9.5	37.4
Consultation topics				
Prescribed drugs including concomitant intake	1.7	52.0	11.3	35.0
Selection of OTC drugs	1.4	48.3	12.8	37.6
OTC drugs including concomitant intake	1.4	48.7	12.6	37.2
Adverse events	1.3	48.9	12.4	37.5
Disease	1.3	43.4	15.6	39.7
Health	1.5	44.0	15.4	39.1
Diet/Nutrients	1.2	38.9	17.2	42.8
Health foods/Dietary supplements	1.2	32.6	23.8	42.4
Interaction between drugs and health food/dietary supplement use	1.1	35.4	22.8	40.7
Adverse events associated with health food/dietary supplement use	1.0	31.6	23.5	43.8
Cessation of smoking/alcohol consumption	0.9	14.9	36.7	47.5
Homecare/Nursing care	0.7	21.0	25.6	52.7
Consultation outside of office hours	1.0	22.4	27.3	49.3
Others	6.0	30.0	20.0	44.0

Differences among groups were examined using the chi-square (*χ*^2^) test.

**Table 8:** Individuals with whom participants wish to consult with regarding each topic.

	Pharmacists	Dieticians	AS ^1^	Anybody/Do Not Care
Selection of OTC drugs	64.6	3.8	4.7	31.8
OTC drugs including concomitant intake	63.0	4.8	4.5	32.0
Adverse events associated with drugs	61.9	5.5	6.2	31.8
Disease	53.1	6.0	9.6	37.8
Health	39.4	20.4	13.3	40.2
Diet/Nutrients	14.2	49.5	8.5	37.6
Health foods	26.8	20.1	17.9	46.6
Interaction between drugs and health foods	33.7	17.8	15.6	45.3
Adverse events associated with health foods	31.1	17.6	16.6	46.0
Cessation of smoking/alcohol consumption	14.9	11.5	13.2	65.4
Homecare/Nursing care	17.4	7.4	12.8	68.4
Consultation outside of office hours	15.5	8.0	21.1	61.9
Others	34.0	22.0	20.0	44.0

^1^ AS: Advisory staff for health foods.

## Data Availability

The data presented in this study are available on request from the corresponding author.
